# Artificial intelligence in planned orthopaedic care

**DOI:** 10.1051/sicotj/2024044

**Published:** 2024-11-21

**Authors:** Elena Chiara Thalia Georgiakakis, Akib Majed Khan, Kartik Logishetty, Khaled Maher Sarraf

**Affiliations:** 1 Cambridge University Hospitals NHS Foundation Trust Cambridge United Kingdom; 2 Imperial College Healthcare NHS Trust London United Kingdom

**Keywords:** Artificial intelligence, Machine learning, Deep learning, Planned orthopaedic care

## Abstract

The integration of artificial intelligence (AI) into orthopaedic care has gained considerable interest in recent years, evidenced by the growing body of literature boasting wide-ranging applications across the perioperative setting. This includes automated diagnostic imaging, clinical decision-making tools, optimisation of implant design, robotic surgery, and remote patient monitoring. Collectively, these advances propose to enhance patient care and improve system efficiency. Musculoskeletal pathologies represent the most significant contributor to global disability, with roughly 1.71 billion people afflicted, leading to an increasing volume of patients awaiting planned orthopaedic surgeries. This has exerted a considerable strain on healthcare systems globally, compounded by both the COVID-19 pandemic and the effects of an ageing population. Subsequently, patients face prolonged waiting times for surgery, with further deterioration and potentially poorer outcomes as a result. Furthermore, incorporating AI technologies into clinical practice could provide a means of addressing current and future service demands. This review aims to present a clear overview of AI applications across preoperative, intraoperative, and postoperative stages to elucidate its potential to transform planned orthopaedic care.

## Introduction

Artificial intelligence (AI) refers to the application of computer algorithms that provide machines with the ability to perform tasks innately characteristic of human intelligence [1]. It encompasses various subfields, including machine learning (ML), deep learning (DL), and natural language processing (NLP) [1]. Grounded in principles of pattern recognition and iterative improvement through training on large datasets, these algorithms can evolve from initially requiring human input to functioning autonomously [2].

Musculoskeletal conditions are the most significant contributor to global disability, with approximately 1.71 billion people afflicted [3]. As such, current service demand for planned orthopaedic surgical procedures is straining healthcare systems globally, exacerbated by the COVID-19 pandemic and the effects of an ageing population [4, 5]. Patients face prolonged waiting times for necessary treatment, resulting in further deterioration and potentially poorer clinical outcomes when they eventually progress to surgery [6].

**Figure 1:**
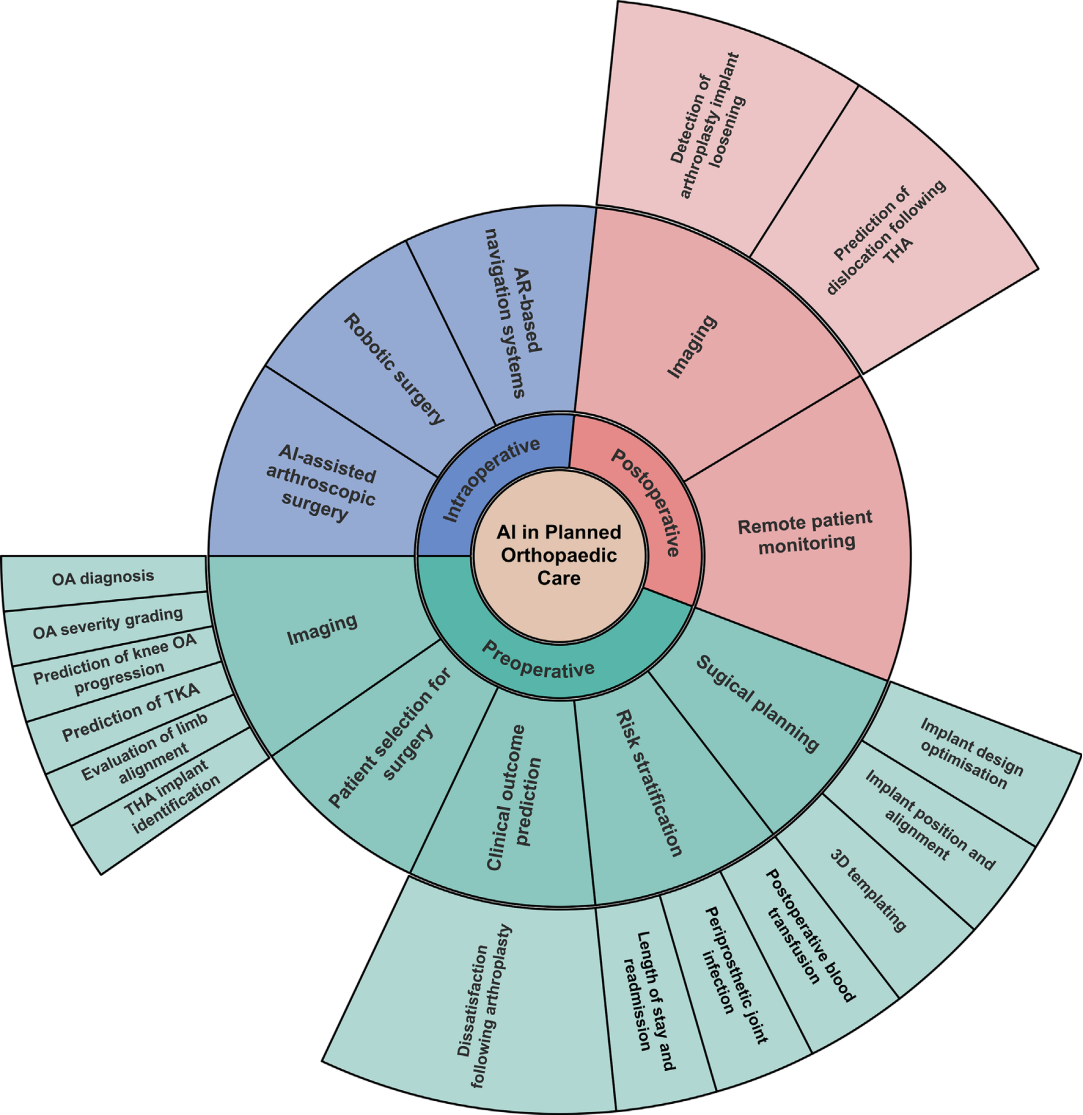
The applications of artificial intelligence in planned orthopaedic care discussed in this article. AI = artificial intelligence, OA = osteoarthritis, TKA = total knee arthroplasty, THA = total hip arthroplasty, NLP = natural language processing, EPR = electronic patient records, PROM = patient-reported outcome measure,, AR = augmented reality.

AI holds promise in transforming planned orthopaedics with wide-ranging applications throughout the perioperative period, encompassing automated diagnostic imaging, clinical decision-making tools, optimisation of implant design, robotic surgery, and remote patient monitoring [1]. Incorporation of these tools into the clinical setting could optimise patient care and streamline system efficiency and thus potentially reduce waiting lists. However, despite the ever-increasing body of orthopaedic research in AI, challenges remain, and current application in clinical practice is limited [7, 8]. This review explores AI’s role in planned orthopaedic care by examining applications across preoperative, intraoperative, and postoperative stages (Figure 1).

## Preoperative

AI is poised to play a pivotal role preoperatively in diagnostic imaging, patient selection, clinical outcome prediction, risk stratification, and surgical planning.

### Imaging

Traditionally, osteoarthritis (OA) diagnosis and classification has relied upon manual radiographic assessment; however, multiple AI algorithms capable of automated detection and staging have now been developed [9–12]. Xue et al. [9] created a model to diagnose hip OA by training a previously validated convolutional neural network (CNN) for image recognition on 420 hip radiographs. The model demonstrated promising diagnostic aptitude, with a performance comparable to that of a physician with ten years of experience [9]. Beyond diagnosis, AI can further categorise the severity of knee OA using the Kellgren-Lawrence (KL) grading system with good accuracy and improved reproducibility compared to manual evaluation [11, 12]. Uniquely, one study using a Deep Siamese CNN architecture provided probability distributions for each KL grade, which may be of clinical utility in grading ambiguous cases [13]. However, it’s important to note that when an algorithm is trained on radiographs from a single institution, generalisability may be encumbered by different labelling systems, radiation exposure and protocols, thus making it invalid [14].

In the context of total joint replacement (TJR), which constitutes a significant portion of planned orthopaedic procedures, a growing body of research has demonstrated the potential of ML and DL models capable of outperforming traditional logarithmic regression (LR) to predict structural disease progression of OA and identify individuals at high risk of TJR [15–19]. Guan et al. [15] developed a DL programme using baseline knee radiographs to predict medial joint space loss over 48 months. The study compared a traditional model incorporating demographic and radiographic risk factors with a DL model trained on 2,300 radiographs from the Osteoarthritis Initiative. The DL model demonstrated significantly higher accuracy, with an AUC of 0.799 compared to 0.660 of the traditional model [15]. Similarly, Leung et al. [18] found that applying DL to predict total knee arthroplasty (TKA) based solely on knee radiographs outperformed conventional binary outcome models, attaining high sensitivity and specificity, 83% and 87%, respectively [13]. Despite the efficacy of these models, there is a well-documented discrepancy between radiologic severity and clinical symptoms in OA; consequently, the decision for TKA is primarily based on symptom severity rather than radiographic findings alone [13, 20, 21]. Therefore, although ML algorithms that automate OA grading or predict arthroplasty based solely on imaging have limited clinical utility, they provide valuable insights for developing more comprehensive predictive tools. Future studies incorporating additional clinical parameters alongside imaging data may yield more clinically relevant tools to support surgical decision-making [13].

A further application of AI-automated pathology quantification lies in the accurate evaluation of limb alignment, which is crucial for planning corrective surgeries for malalignment and joint replacement. Lee et al. [22] trained and tested a DL-based algorithm to quantify four alignment parameters of the knee; on external validation, not only did the measurement angles strongly correlate with those performed manually, but it was also 3.44 times faster. Similarly, in the context of preoperative planning for TKA, Vidhani and colleagues validated a DL and computer vision-based framework for measuring five hip and knee anatomic parameters from CT scans; the average time taken was 2.58 (±1.92) minutes per patient with no significant difference from manual measurements [23]. Central to both systems is the incorporation of CNNs for automatic image segmentation, precisely identifying the specific regions of interest from which measurements are derived [14].

As the number of total hip arthroplasty (THA) procedures increases, revisions are anticipated to rise by between 43% to 70% by 2030 [24]. Preoperative planning involves correctly identifying the implant type; this can be difficult and time-consuming, typically relying on radiographs, medical records, and implant labels. Although numerous AI-powered software programs have been developed to identify implants automatically, they often face limitations such as small training datasets, training exclusively on AP radiographs, and lack of acetabular classification [25]. Addressing these challenges, Rouzrokh et al. [25] developed a DL tool for THA automatic implant detection, trained on over 240,00 radiographs. It can identify common designs of 20 femoral and 8 acetabular components from various views (AP, lateral, or oblique), achieving an internal accuracy of 98.9% for both components and 97.0% accuracy in external validation for femoral components [25]. To explain model performance, they leveraged gradient mapping to highlight the radiographic features affecting network decisions. A unique aspect of their model is the incorporation of uncertainty quantification (UQ) to gauge the confidence in the model’s output, along with outlier detection to identify data that may compromise results. These features enhance the model’s trustworthiness, supporting its potential for clinical deployment [25].

### Patient selection

Prolonged waiting times adversely impact patients’ preoperative well-being and may detract from the potential gains of arthroplasty surgery [6]. Automated stratification of patients based on routine EPR data through NLP could be employed to guide clinical pathways and streamline the referral process for operative intervention, thus improving system efficiency. NLP enables computers to understand and analyse human language by translating it into structured data, overcoming the limitations of manual processing in terms of timeliness, reproducibility, and scalability [1, 26].

Patients seeking treatment through the UK’s National Health Service (NHS) rely on referrals from non-specialist general practitioners (GPs). Farrow et al. [4] are exploring the use of AI to assist GPs in correctly and rapidly identifying suitable candidates for arthroplasty surgery as part of the AI to Revolutionise the Patient Care Pathway in Hip and Knee Arthroplasty (ARCHERY) project. The study hinges on employing NLP to synthesise patient information, such as imaging reports, clinical letters, patient health records, and patient-reported outcome measures (PROMs), to predict if a patient would be suitable for surgery. Preliminary results discussed at the British Hip Society meeting in February 2024, focusing on their THA model, indicated that NLP fails to perform accurate clinical inference for identifying patients appropriate for THA, with performance deteriorating further on external validation [27]. This highlights the challenge of applying NLP across different healthcare settings or globally, where heterogeneity of clinical terminology, free text information, and criteria for surgical candidacy exist [28].

### Clinical outcome prediction

PROMs serve as a quality benchmark in arthroplasty. As many as 20–30% of patients who undergo TJR report no improvement or dissatisfaction with their surgical results [29, 30]. Identifying patients unlikely to achieve long-term, meaningful gains from surgery is crucial for setting patient expectations and informing presurgical decision-making, which could be advantageous in reducing costs to healthcare systems by preventing patients unlikely to benefit from receiving unnecessary treatment [2, 13].

Multiple ML models have been developed to predict patient dissatisfaction, offering potentially valuable adjuncts to support shared decision-making [13, 31–34]. When high-risk patients are recognised, interventions such as preoperative education and health optimisation may be implemented, while others may choose to seek non-surgical options [34, 35]. Liu et al. [33] trained three ML models (image-only, clinical-data-only, and multimodal) on a cohort of 5,720 knee OA patients to predict postoperative dissatisfaction at two years. Dissatisfaction was defined as failing to achieve the minimal clinically important difference (MCID) – the minimum change considered beneficial or meaningful – corresponding to three PROM scoring systems, including the Oxford Knee Score (OKS). The clinical-data-only and multimodal models demonstrated excellent performance, with AUCs of 0.806 and 0.816, respectively. Preoperative functional scores and mental health status were among the most influential features correlating with dissatisfaction [33].

Using PROMs as a metric for patient dissatisfaction or attempting to quantify a subjective measure for AI prediction has inherent limitations. Challenges lie in their inability to capture the nuanced outcomes of surgery, compounded by the ceiling effect whereby patients who are relatively well before surgery may show modest measurable improvement postoperatively, as the potential for significant change is constrained [31]. Hunter and colleagues demonstrated this in their study, where an unsupervised ML programme identified clusters of patients with predictable PROM changes [31]. Those with the worst preoperative scores experienced the greatest improvements, conversely, patients who were relatively well, or whose quality of life was less affected preoperatively, did not show as marked a change in PROM despite their clinical outcomes being adequate. Suggesting that while AI models can be useful in predicting dissatisfaction, they may be less effective in predicting outcomes for those with less severe baseline symptoms. Therefore, relying solely on PROM scores to generate AI models could oversimplify the complexities of patient experiences, underscoring the need for more comprehensive and personalised tools in assessing surgical outcomes.

### Risk stratification

AI-based algorithms that predict transfusion risk, periprosthetic joint infection (PJI), length of stay (LOS), and unplanned readmission could be used to forecast patient outcomes following orthopaedic interventions [36–44]**.** These models can assist in preoperative optimisation, resource management, and treatment planning, improving patient care and healthcare efficiency.

Jo et al. [38] conducted the first study leveraging ML to predict blood transfusion after TKA using six preoperative variables (haemoglobin, platelet count, type of surgery, tranexamic acid use, age, and body weight). The model demonstrated good performance with an AUC of 0.880 on external validation [38]. Conversely, in their study, Harris et al. [37] found that while models performed with good accuracy at discriminating the likelihood of patients experiencing renal or cardiac complications and death within 30 days of arthroplasty, on external validation, they were no longer robust in predicting renal complications, demonstrating the need for further development prior to clinical adoption. They postulated that incorporating NLP analysis of clinical progress notes and radiographs could be used to improve predictive accuracy [37].

PJIs represent a severe complication of TJA, leading to increased morbidity, readmissions, and substantial healthcare costs [45]. Predicting PJI accurately is challenging due to the numerous patient and surgical risk factors [46]. AI could be deployed clinically to improve PJI prevention and management by assessing risk and assisting in diagnosis, antibiotic selection, and prognosis [47–50]. Yeo et al. [48] developed an artificial neuronal network to predict superficial surgical site infections and PJIs following TKA. The model was trained on 10,021 patients with an average follow-up of 2.8 years and found predictors included obesity, Charlson Comorbidity Index, and smoking, with obesity emerging as a stronger predictor of infection than previously thought, possibly due to the increased accuracy of data analysis provided by ML algorithms in handling complex datasets [48]. Furthermore, an ML system outperformed the widely used International Consensus Meeting (ICM) criteria for diagnosing PJI [49, 51]. Unlike the fixed ICM criteria, which relies on factors that may be infrequent or expensive to assess, ML can construct adaptive diagnostic models based on available data, incorporating explainability features such as “if-then” decision paths to increase transparency and clinician trust [49].

LOS is the principal cost driver in the episode of care, and reliable predictions could improve resource allocation and care equity, particularly when integrated into payment models that account for patient-specific risks rather than using a “one-size-fits-all” approach [39, 52]. Ramkumar et al. [39] devised an algorithm to predict payment based on LOS; however, as patient complexity increased, the error margin in payment prediction also grew, reaching 32% for patients with extreme comorbidities, underscoring the difficulties of managing complex cases in payment models [39]. Nevertheless, a key benefit of ML models is their ability to capture nonlinear relationships that traditional analytical methods may overlook, offering insight into the factors influencing outcomes. For example, in their LOS predictive model, Chen et al. [41] identified age, transfusion, BMI, operation time, and preoperative blood results (haematocrit, platelet count, and white blood cell count) as the most important predictors of LOS. In another study, NSAID use, systemic corticosteroids, and poor social support were significant predictors of readmission after TJR [43]. Based on such findings, clinicians may be able to allocate additional resources to target high-risk individuals more effectively.

However, using such predictive methods engenders concerns about the potential marginalisation of patients deemed too high risk who may be labelled as inoperable. Therefore, careful consideration is needed to ensure these models are applied equitably and do not exclude vulnerable individuals.

### Surgical planning

Preoperative planning AI software can inform implant design, positioning, and 3D templating [53–56]. For example, Cilla et al. [53] applied ML to optimise short stem implant design to reduce stress shielding, while Jang and colleagues used DL to estimate patient-specific adjustments for optimal landmark usage in TKA planning [54].

In their retrospective review, Ding et al. evaluated the accuracy of an AI-based 3D preoperative planning software (AIHIP; Beijing Changmugu Medical Technology Co., Ltd., Beijing, China) for THA [55]. They found that it offered a more accurate surgical planning tool than traditional 2D templating by enabling precise visualisation of anatomical structures, facilitating reliable predictions of component size and implant position, surpassing the accuracy of manual techniques [55]. This leaves surgeons better prepared for surgery, helping to avoid unforeseen intraoperative challenges and reducing the time required for preoperative planning. That said, applying this technology incurs additional costs and radiation exposure, as it requires preoperative CT scans. Future AI applications may mitigate these drawbacks; for instance, Fernandes et al. [57] have developed an innovative AI program that can convert 2D radiographs into 3D bone models with high accuracy, reliability, and repeatability compared to CT scans.

## Intraoperative

The integration of AI intraoperatively is rapidly expanding, with its incorporation into tools such as augmented reality (AR)-based navigation systems, AI-assisted arthroscopic procedures, and robotic surgery [36]. These innovations aim to support decision-making, minimise the risk of complications, and reduce inter-surgeon variability to deliver consistent and high-quality care between institutions [36, 58]. For instance, a navigation-assisted anchor screw insertion tool for shoulder arthroscopy has shown high repeatability and reproducibility in cadaveric studies, with both novice and expert operators attaining insertion of the anchor at an angle close to the predetermined target angle [58].

Robotic surgery, known for its minimally invasive approach coupled with greater precision, has been demonstrated to reduce blood loss, pain, and recovery time [59, 60]. AI’s role in this field is expanding with the scope for the integration of ML algorithms capable of refining surgical planning through data analysis from previous surgeries [36]. Additionally, real-time feedback during procedures involving bone cutting or soft tissue resection can enhance precision and reduce the likelihood of errors, thereby ensuring consistent and reproducible results [36]. Nonetheless, the widespread adoption of robotics into clinical practice faces various challenges. These include the substantial financial investment to acquire and maintain the equipment, the consumables used during procedures, system compatibility with only specific implant types, and the need for surgeon and staff training to ensure safe use [36].

As these technologies become increasingly integrated into modern practice, there are concerns regarding automation bias resulting in overreliance on technology. This could detract from surgeons’ clinical skills and, if technology fails, lead to severe consequences. Drawing parallels with the airline industry’s use of autopilot systems, it forces speculation as to whether surgeons may be primarily only called upon in emergencies should things go wrong. Moreover, it raises the question of whether surgical training should evolve to include knowledge and operation of these machines.

## Postoperative

Critical to the success of surgery is the postoperative recovery phase, rehabilitation, and monitoring for complications. Through wearable devices and automated analysis of post-operative imaging, AI could be used to enhance this.

### Remote patient monitoring

Internet of Things and mHealth refers to incorporating AI into wearable devices, sensors, and smartphone apps that can track various parameters and collect large volumes of real-time data, which clinicians can monitor to assess rehabilitation progress and intervene if milestones are unmet [1, 36]. Such innovations have shown value in reducing opioid use and improving therapeutic adherence with anticoagulation for venous thromboembolism prophylaxis [61, 62].

Ramkumar et al. [63] validated a motion-based ML software kit to assess arcs of shoulder motion. They applied this technology to devise a system for remote patient monitoring of mobility, home exercise compliance, and range of motion following TKA [64]. The system comprises a knee sleeve containing two Bluetooth sensors that transmit three-dimensional spatial orientation changes to a smartphone app. The app processes the data using the ML algorithm, while also collecting additional information entered by the patients, such as weekly PROMs and opioid consumption. Overall, it showed promise, with patients finding it motivating and engaging, and allowed mobility and compliance with physiotherapy to be evaluated remotely, potentially alerting clinicians to early complications [64].

### Image analysis to screen for complications

The utility of AI in image analysis extends beyond the preoperative period; applications have also been tested postoperatively to detect early signs of implant loosening and determine the risk of dislocation with greater accuracy and efficiency than traditional methods [65, 66]. Through early detection, prompt intervention may be sought.

Prosthetic loosening is the leading cause of arthroplasty failure, accounting for 30% and 55% of revision surgeries following TKA and THA, respectively [67, 68]. Borjali et al. [65] developed a fully automated CNN algorithm to detect mechanical loosening of THA implants from plain film radiographs, reporting a lower specificity of 75.0% but a higher sensitivity of 91.6% compared to manual assessment by an expert orthopaedic surgeon, illustrating its potential role in supplementing the decision-making process, freeing time for clinicians to engage in more patient-centric activities.

Similarly, dislocation following THA presents a considerable challenge, resulting in severe pain, limb dysfunction, readmission, revision, and substantial financial implications, with treatment costs estimated to be three times higher than those with uncomplicated THA [69]. Identifying patients at risk of dislocation is crucial for tailoring postoperative rehabilitation protocols [70]. A CNN, trained on over 92,000 radiographs, has been developed to classify THA patients for dislocation risk based on single postoperative AP pelvis radiographs, achieving 89.0% sensitivity [66]. Given the burden of follow-up visits and limitations of hip precautions, those identified by CNN as low risk may be managed with fewer postoperative restrictions.

## Conclusion

Incorporating AI-powered technologies across the perioperative period offers an opportunity to improve system efficiency and enhance patient care as a means of addressing the growing strain on planned orthopaedic services. Nevertheless, key challenges such as accuracy, generalisability, and transparency must be addressed. Few models discussed in this review have undergone external validation, and a high AUC does not necessarily equate to strong clinical performance. Furthermore, robust regulatory and quality frameworks must be concurrently created as metrics from which these models can be assessed to ensure safe and effective implementation.

## Data Availability

This article has no associated data generated.
